# Integrative analysis reveals shared biomarkers and pathways in osteoporosis and rheumatoid arthritis using machine learning and single-cell sequencing

**DOI:** 10.1371/journal.pone.0362219

**Published:** 2026-11-04

**Authors:** Jin Zhao, Yixuan Chen, Fei Yang, Cheng Tian, Wenyan Luo, Zhou Su, Bohuan Yang, Wei Liu, Hongbo Yang

**Affiliations:** Department of Orthopedics, Hunan Prevention and Treatment Institute for Occupational Diseases, Affiliated Prevention and Treatment Institute for Occupational Diseases of University of South China, Changsha, Hunan, China; The Affiliated Changzhou No 2 People’s Hospital of Nanjing Medical University, CHINA

## Abstract

**Background:**

Osteoporosis (OP) and Rheumatoid Arthritis (RA) are prevalent chronic diseases characterized by bone loss and immune dysregulation. Evidence suggests that patients with RA are at increased risk of developing OP; however, the shared molecular mechanisms linking these conditions remain incompletely understood.

**Methods:**

Publicly available bulk transcriptomic and single-cell RNA sequencing (scRNA-seq) datasets for OP and RA were obtained from the Gene Expression Omnibus (GEO) database. Weighted gene co-expression network analysis (WGCNA) was applied to identify shared gene modules associated with both diseases. Functional enrichment and protein–protein interaction analyses were performed to characterize common biological pathways. Machine learning algorithms, including Random Forest (RF) and Support Vector Machine (SVM), were used to identify and validate shared hub genes and to construct diagnostic models. Immune cell infiltration was evaluated using CIBERSORT, and the cellular localization of hub genes was further validated using scRNA-seq analysis.

**Results:**

WGCNA identified 329 genes shared between OP- and RA-associated modules, which were significantly enriched in muscle-related processes, cytokine signaling, and immune–inflammatory pathways. RF analysis identified three shared candidate genes: ANO5, IL27RA, and CNTN3. Among these, ANO5 and IL27RA demonstrated consistent expression evidence and literature support, whereas CNTN3 emerged as a computationally nominated candidate requiring cautious interpretation and experimental validation. An SVM-based model incorporating these genes demonstrated robust predictive accuracy in both OP and RA validation cohorts. Immune infiltration analysis revealed distinct yet coordinated immune landscapes, with hub genes showing significant correlations with T cells, macrophages, B cells, and plasma cells. ScRNA-seq analysis revealed that ANO5 was predominantly expressed in macrophages and fibroblasts, IL27RA was mainly enriched in T cells, whereas CNTN3 showed minimal enrichment at the single-cell level in both OP bone and RA synovial tissues.

**Conclusion:**

This integrative bioinformatics and machine learning study identifies shared molecular signatures and immune-related mechanisms underlying OP and RA. ANO5 and IL27RA emerge as robust candidate regulators linking immune dysregulation and bone remodeling, whereas CNTN3—although identified by machine learning—requires cautious interpretation and further investigation due to limited supporting evidence. These findings provide novel insights into the pathogenesis of RA-associated OP and highlight potential biomarkers and therapeutic targets for their comorbidity.

## Introduction

Osteoporosis (OP) is a chronic skeletal disorder marked by decreased bone density and structural degeneration, resulting in heightened fragility and an elevated risk of fractures [1]. Osteoporotic fractures, which predominantly occur in postmenopausal women and older adults, are a prevalent cause of mortality in this population and pose a significant global public health burden [2]. The pathogenesis of osteoporosis is multifactorial and primarily involves disruptions in bone homeostasis, driven by imbalances in hormones, cytokines, and growth factors [3]. Recent studies have identified several regulators of bone metabolism and associated signaling pathways, including BMP/TGF-β [4], Wnt/β-catenin [5], HIF-1α/RANKL/Notch1 [6], and JAK-STAT [7]. Despite extensive research, the precise molecular mechanisms underlying osteoporosis remain incompletely understood, necessitating further investigation.

Rheumatoid arthritis (RA) is a systemic autoimmune syndrome marked by joint inflammation and progressive bone damage [8]. RA is associated with increased morbidity and mortality, from joint destruction but also due to its systemic effects. Cardiovascular complications are among the most significant contributors to poor prognosis and are now recognized as a leading cause of death in RA patients [9]. RA is driven by various risk factors. Non-modifiable risk factors include genetic predisposition and sex, whereas modifiable factors include smoking, dietary habits, and environmental exposures [10]. Studies have reported an increased prevalence of osteoporosis among patients with RA [11], indicating potential shared pathogenic mechanisms, overlapping immunological pathways, and convergent therapeutic strategies between autoimmune and metabolic bone diseases.

Extensive studies have revealed that immune cell activity, inflammatory mediators, and bone remodeling dynamics are intricately interconnected in the pathophysiology of both RA and osteoporosis [12–14]. Chronic inflammation in RA could disrupt skeletal remodeling homeostasis, leading to RA-associated bone loss. Synovial fibroblasts and immune cells further aggravate this process by promoting RANKL-dependent osteoclast genesis and activating inflammatory pathways, thereby accelerating bone erosion and increasing fracture risk [15]. Therefore, investigating the molecular links between RA and osteoporosis holds substantial research value and therapeutic significance.

In this study, we conducted an integrative bioinformatics analysis incorporating machine learning algorithms to identify shared hub genes and molecular pathways underlying RA and OP using publicly available datasets from the Gene Expression Omnibus (GEO) database. Furthermore, we explored the correlation between these hub genes and immune cell infiltration, as well as key metabolic pathways relevant to both diseases. Finally, we validated the expression patterns and spatial distribution of the most significant hub genes using scRNA-seq analysis.

## Materials and methods

### Data selection

The keywords “osteoporosis” (OP) and “rheumatoid arthritis” (RA) were used to search for gene expression profiles in the GEO database. For OP, the gene expression proﬁling by array datasets GSE35959 and GSE230665, along with the single-cell RNA sequencing dataset GSE147287, were downloaded from the GEO. GSE35959 includes 5 OP samples and 14 healthy controls, GSE230665 contains 12 OP samples and 3 controls, and GSE147287 comprises single-cell data from 1 OP patient. For RA, datasets GSE77298 and GSE93272, along with the single-cell sequencing dataset GSE200815, were downloaded from the GEO. GSE77298 includes 16 RA samples and 7 healthy controls, GSE93272 contains 229 RA samples and 46 controls, and GSE200815 comprises data from 4 RA patients.

The demographic data of samples in these data sets is provided in S1 Table.

### Construction of weighted gene co−expression network analysis

Weighted gene co-expression network analysis (WGCNA) analysis was conducted on the GSE35959 and GSE77298 datasets to identify gene modules, utilizing the “pickSoftThreshold” function from the WGCNA package [16]. Genes ranked in the top 5000 by their median absolute deviation (MAD) in the expression matrix were selected for the analysis (corresponding to MAD > 0.35 for GSE35959 and MAD > 0.42 for GSE77298). After filtering out genes with zero variance and missing values, the remaining data were used to construct an adjacency matrix according to the scale-free topology criterion (scale-free R^2^ = 0.9). This matrix was subsequently converted into a topological overlap matrix (TOM). Gene modules were then defined using the average-linkage hierarchical clustering method, grouping genes with similar expression patterns. To determine the key modules, a minimum module size of 30 was specified, and a cut height of 0.2 was applied. The grey module represented genes that could not be classified into any specific module. Finally, a Pearson correlation analysis was performed to assess the relationship between the identified modules and the traits associated with OP and RA.

### Pathway and functional enrichment analysis

Feature biomarkers were identified at the intersection of the WGCNA analysis. Metascape (http://metascape.org/gp/index.html#/main/step1) was used to perform Gene Ontology (GO) term enrichment analysis and Kyoto Encyclopedia of Genes and Genomes (KEGG) pathway analysis, in order to pinpoint the pathways where the feature genes were significantly enriched in both OP and RA [17]. The criteria for selecting significantly enriched GO terms and KEGG pathways were a minimum overlap of three and a minimum enrichment value of 1.5. The significance of enrichment was considered when the adjusted p-value was below 0.05.

### Hub genes screening and validation based on the machine learning algorithm

To identify hub genes from the gene set intersecting in the WGCNA results, the random forest (RF) algorithm [https://doi.org/10.1023/A:1010933404324] was employed. A classification model was built using RF, based on the gene expression data from the training cohort. Feature importance was evaluated based on the Mean Decrease in Gini, and the top 30 significantly contributing genes were visualized using the ‘ggplot2’ package in R. Subsequently, the model’s predictive performance was evaluated using the validation dataset, and its effectiveness was quantified by calculating the area under the receiver operating characteristic curve (AUC).

### Construction of the diagnostic model based on the hub genes

The Support Vector Machine (SVM) classifier was constructed using the e1071 package in R, with the expression levels of the hub genes serving as input features for model training. All gene expression values were standardized to zero mean and unit variance prior to SVM training. An RBF kernel was used, with the cost parameter (C) searched over 2^−5^ to 2^5^ and gamma over 2^−5^ to 2^5^ via 10‑fold cross‑validation on the training data. The optimal parameters were C = 2 and gamma = 0.5 for the OP model, and C = 4 and gamma = 0.25 for the RA model. For OP, GSE35959 was employed as the training dataset, while GSE230665 was used for external validation. For RA, GSE77298 served as the training dataset, and GSE93272 was used as the validation cohort. To minimize the risk of overfitting and information leakage, all feature selection and model training procedures were performed exclusively within the training datasets; the external validation datasets were completely sequestered and not accessed until the final model evaluation stage. No feature selection or hyperparameter tuning was performed using the validation datasets, and the validation sets were used only once—at the final step—to assess the predictive performance of the fully trained models. The predictive performance of the SVM model was evaluated using receiver operating characteristic (ROC) and precision-recall (PR) curves, along with their respective AUC values.

### Correlation analysis of hub gene expression with immune infiltration

CIBERSORT, a widely adopted deconvolution method, was employed to estimate the abundance of 22 distinct immune cell types within the microenvironment of the GSE35959 and GSE77298 datasets [18]. The analysis was performed using the LM22 signature matrix with 1,000 permutations, and samples with CIBERSORT p < 0.05 were considered to have reliable deconvolution results and were retained for downstream analysis. To explore the relationship between hub genes and immune cell infiltration, Spearman correlation analysis was performed. The results were visualized using the ggplot2 and pheatmap packages in R.

### Single-cell data processing and clustering

For single-cell sequencing analysis, the raw data for the OP dataset GSE147287 and the RA dataset GSE200815 were downloaded from the GEO database. The raw data were initially processed using the Seurat R package under the R programming environment. Rigorous quality control criteria were applied to ensure data integrity: specifically, cells with fewer than 200 detected genes were filtered out; potential doublets or low-quality cells were removed by restricting the number of genes to a reasonable range; and cells where the mitochondrial gene percentage exceeded 20% were filtered out. For samples containing an extremely large number of cells, a representative subset of 10,000 cells was retained via random down-sampling to optimize computational efficiency for downstream analysis. The QC-filtered data were then normalized using the LogNormalize method to stabilize variance, followed by the identification of 3000 Highly Variable Features (HVFs). Principal Component Analysis (PCA) was performed on the expression matrix of these HVFs to reduce dimensionality, retaining the top 50 Principal Components (PCs) for subsequent steps. Cell clustering was achieved by constructing a K-Nearest Neighbor (KNN) graph based on the top 10 PCs, to which the Louvain algorithm was applied for modularity optimization, employing a Resolution of 0.3. Non-linear dimensionality reduction for visualization was performed using the uniform manifold approximation and projection (UMAP) algorithm. Cell types were automatically annotated using the SingleR R package. This annotation was conducted at the cluster level by comparing the mean gene expression profile of each cluster against the reference data using the Wilcoxon rank-sum test to determine the final cell type labels. Finally, the expression patterns of key marker genes, including CNTN3, ANO5, and IL27RA, were visualized using Feature plots, and Dot plots.

## Results

### Weighted gene co-expression network analysis of OP and RA

We performed WGCNA to explore the correlation between clinical traits and genes. All samples were clustered in the GSE35959 and GSE77298 datasets, and none of the samples was eliminated (Fig 1A, 1B). According to the WGCNA methodology, the optimal soft-power value for GSE35959 was 5 while for GSE77298 it was 7 (Fig 1C, 1D). A total of 11 modules were identified in GSE35959, and 11 were identified in GSE77298. Afterwards, the correlations between the module and clinical traits were calculated. The black, grey60 and red modules had the strongest relation with OP (r = 0.58, 0.56 and 0.68) in the GSE35959 database (Fig 1G). For RA, the green, red and brown modules showed the strongest correlation (r = 0.65, 0.65 and 0.66) in the GSE77298 database (Fig 1H). A total of 329 overlapped module genes of GSE35959 and GSE77298 were observed (Fig 1I).

**Fig 1:**
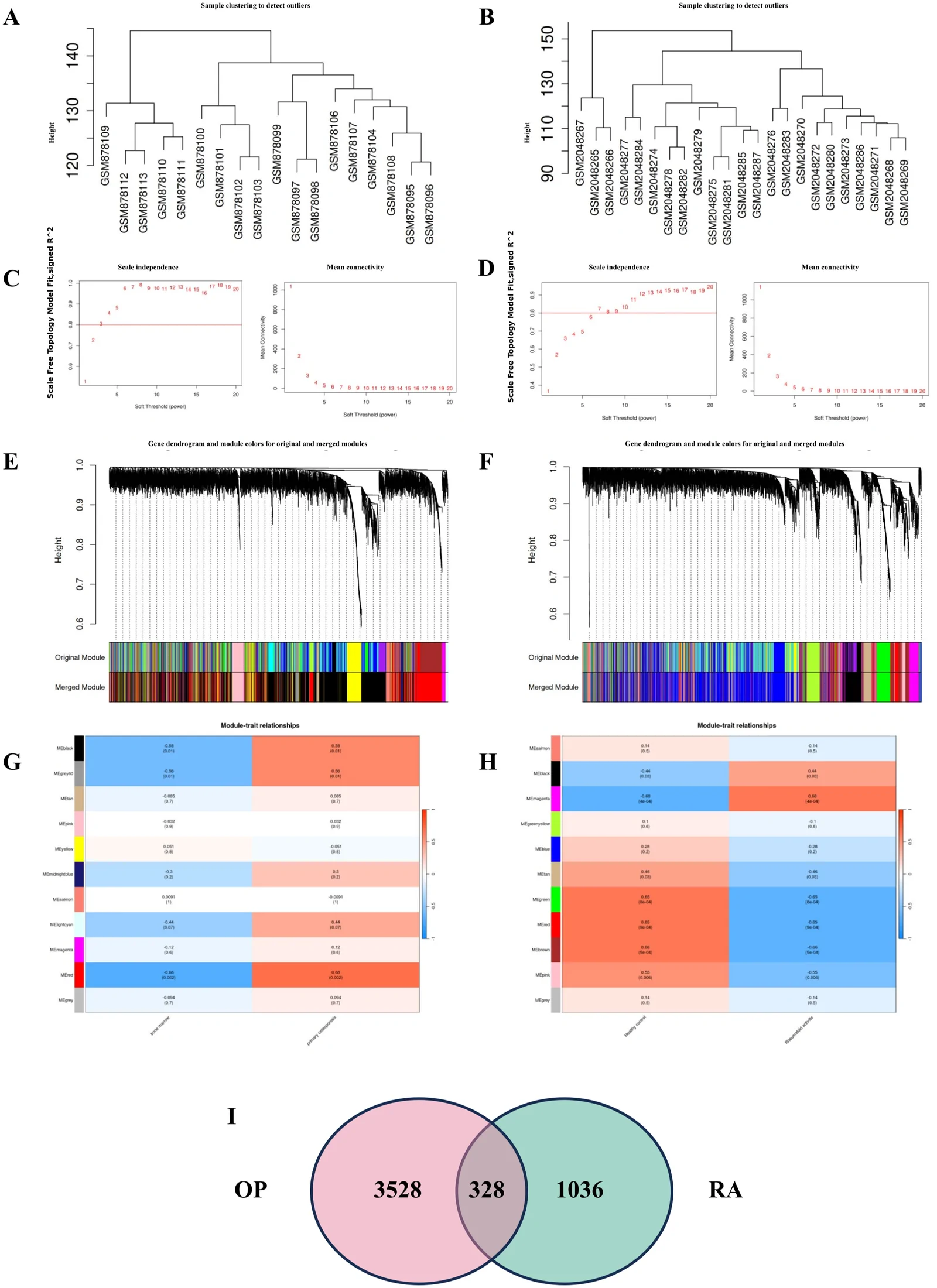
WCGNA analysis of OP and RA. (A) Clustering according to the expression level of OP patients in GSE35959. (B) Clustering according to the expression level of RA patients in GSE77298. (C) Determination of Soft Threshold power for GSE35959. (D) Determination of Soft Threshold power for GSE77298. When scale-free distribution is reached, the optimal soft-power value for GSE35959 was 5 while for GSE77298 it was 7. (E) Origin and merged modules displaying under the clustering tree for GSE35959. (F) Origin and merged modules displaying under the clustering tree for GSE77298. Cluster dendrograms showed the clustering process of the gene modules (G) Heatmap of the correlation between module eigengenes and the occurrence of OP. (H) Heatmap of the correlation between module eigengenes and the occurrence of RA. (I) Overlapping module genes of GSE35959 and GSE77298.

WGCNA was conducted to investigate the relationship between gene expression patterns and clinical traits. Sample clustering in the GSE35959 and GSE77298 datasets confirmed that all samples met quality control criteria, and none were excluded from further analysis (Fig 1A, 1B). Based on scale-free topology criteria, the optimal soft-thresholding power was determined to be 5 for GSE35959 and 7 for GSE77298 (Fig 1C, 1D). In these two datasets, 11 co-expression modules were identified, respectively. Module–trait correlation analysis revealed that in GSE35959, the black, grey60, and red modules were most strongly associated with osteoporosis (r = 0.58, 0.56, and 0.68, respectively; Fig 1G). In GSE77298, the green, red, and brown modules exhibited the highest correlation with rheumatoid arthritis (r = 0.65, 0.65, and 0.66, respectively; Fig 1H). Cross-referencing the module genes from both datasets identified 329 overlapping genes (Fig 1I), which were considered potential candidates involved in shared disease mechanisms.

### Enrichment analysis and PPI network construction

A total of 329 genes were identified at the intersection of the WGCNA modules common to both OP and RA, suggesting their potential involvement in the shared pathogenic mechanisms of these conditions. To study the biological functions and signaling pathways associated with these overlapping genes, comprehensive functional enrichment analyses were conducted, including GO and KEGG enrichment analysis.

The results demonstrated that the 329 genes were significantly enriched in several muscle-related biological processes and pathways, including muscle contraction, muscle organ development, and regulation of skeletal muscle contraction. In addition, several immune and inflammation associated pathways were identified, such as cytokine–cytokine receptor interaction, rheumatoid arthritis, inflammatory response, and viral myocarditis (Fig 2A). Further biological process analysis based on the GO enrichment analysis revealed that these genes were involved in broader functional categories, such as multicellular organismal process, developmental process, regulation of biological processes, and immune system process (Fig 2B).

**Fig 2:**
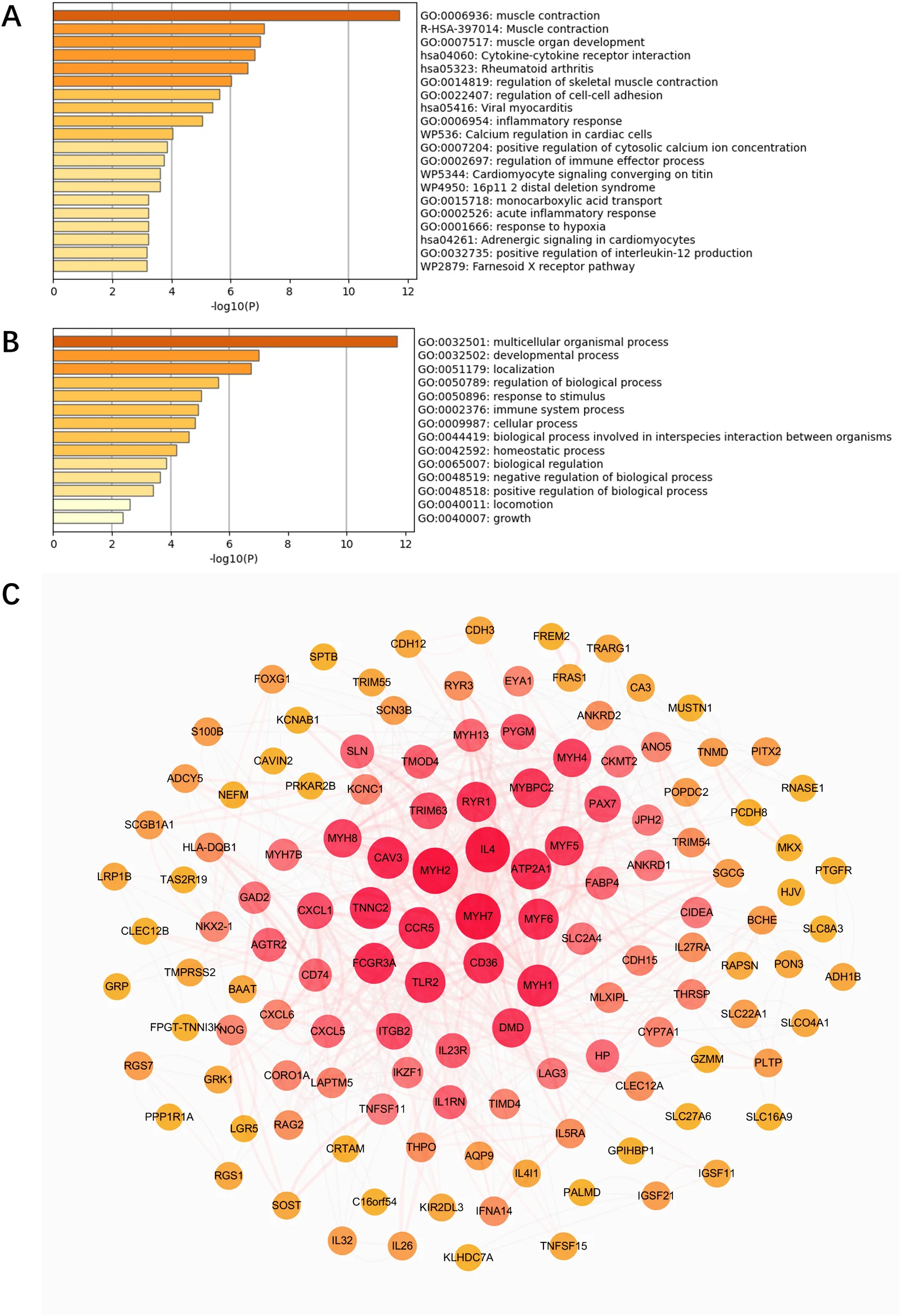
Functional enrichment and PPI network analysis of the 329 shared genes. (A) Top 20 significantly enriched clusters derived from the feature genes, with color intensity corresponding to p-values. (B) Enriched top-level Gene Ontology biological processes, colored by p-values; darker shades indicate stronger pathway enrichment. (C) PPI network of the feature genes. Node size and color depth reflect degree centrality within the network; larger nodes with darker colors indicate greater importance.

Additionally, a protein–protein interaction (PPI) network was constructed via the STRING database, applying a stringent combined interaction score threshold of >0.8 (Fig 2C). The resulting network comprised 233 nodes and 375 edges (S1 Fig). The top5 significant genes with the highest ranking (S2 Fig) were MYH7(Myosin Heavy Chain 7), MYH2(Myosin Heavy Chain 2), IL4(Interleukin-4), TNNC2(troponin C2, fast skeletal type Gene), and MYH1(Myosin Heavy Chain 1).

### Identification and validation of potential shared hub genes by random forest

To further screen the hub genes with the most diagnostic values in “OP” and “RA,” we selected the foremost characteristics based on machine-learning algorithms. Random Forest analysis was carried out. The Random Forest models were successfully built, with error rates stabilizing after approximately 200 trees, indicating good model convergence (Fig 3A, 3B). Fig 3C and 3D showed the top 30 significant genes for the “OP” and “RA” models according to MeanDecreaseGini rank. By intersecting the Top 30 genes ranked by MeanDecreaseGini from the OP RF model (GSE35959) and the RA RF model (GSE77298), we identified 3 shared candidate genes: ANO5, IL27RA, and CNTN3 (Fig 3E). No additional filtering criteria were applied beyond this direct intersection, which were considered to carry the maximum diagnostic value (Fig 3E).

**Fig 3:**
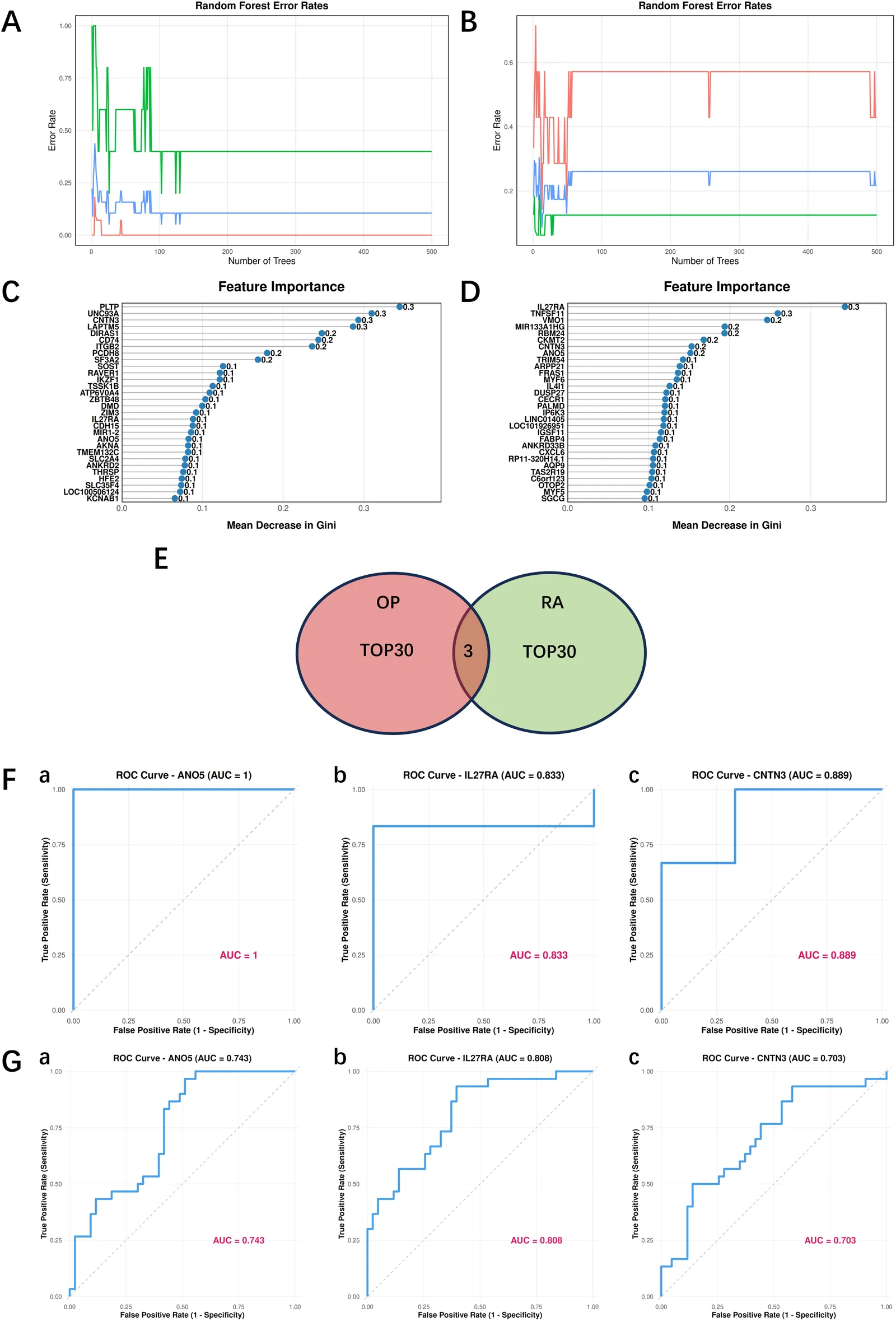
Identification of shared candidate genes via RF and evaluation of their discriminative ability using SVM. (A) Random forest analysis of the GSE35959 dataset for OP. (B) Random Forest analysis of the GSE77298 dataset for RA. (C) Feature importance ranking of OP-associated genes from the RF model based on the GSE35959 dataset. MeanDecreaseGini showed the rank of genes in accordance with their relative importance. (D) Feature importance ranking of RA-associated genes from the RF model based on the GSE77298 dataset. (E) Venn diagram showing the intersection of the Top 30 genes ranked by MeanDecreaseGini from the OP RF model (GSE35959) and the RA RF model (GSE77298). Three overlapping candidate genes (ANO5, IL27RA, CNTN3) were identified. (F) SVM–based ROC curve analysis evaluating the diagnostic performance of the 3 hub genes for OP. (G) SVM–based ROC curve analysis evaluating the diagnostic performance of the 3 hub genes for RA.

### Construction of diagnostic model based on SVM

Moreover, we validated the diagnostic efficacy of these three shared hub genes through ROC curve analysis (Fig 3F, 3G) via the SVM, a widely adopted algorithm known for effectively managing high-dimensional biological datasets. In the OP model, the AUC values for the three genes were ANO5 (AUC = 1.000), IL27RA (AUC = 0.833), and CNTN3 (AUC = 0.889); in the RA model, their respective AUC values were ANO5 (AUC = 0.743), IL27RA (AUC = 0.886) and CNTN3 (AUC = 0.763). While each hub gene holds potential as a supplementary marker for diagnosis or prognosis, our goal was to build a diagnostic model to improve classification precision and predictive power. We thus adopted a machine learning strategy to examine whether the selected hub genes could establish a robust diagnostic model.

To test the generalizability and external validity of this model in OP, GSE35959 was designated as the training dataset, and GSE230665 functioned as the independent validation cohort. The SVM model was constructed using a radial basis function (RBF) kernel, with key parameters (e.g., cost and gamma) optimized through 10-fold cross-validation on the training data. Evaluation on the external validation dataset employed both the ROC and PR curves. The corresponding AUC were 0.946 and 0.784, respectively (Fig 4A, 4B), underscoring the model’s high accuracy and strong capacity for generalization.

**Fig 4:**
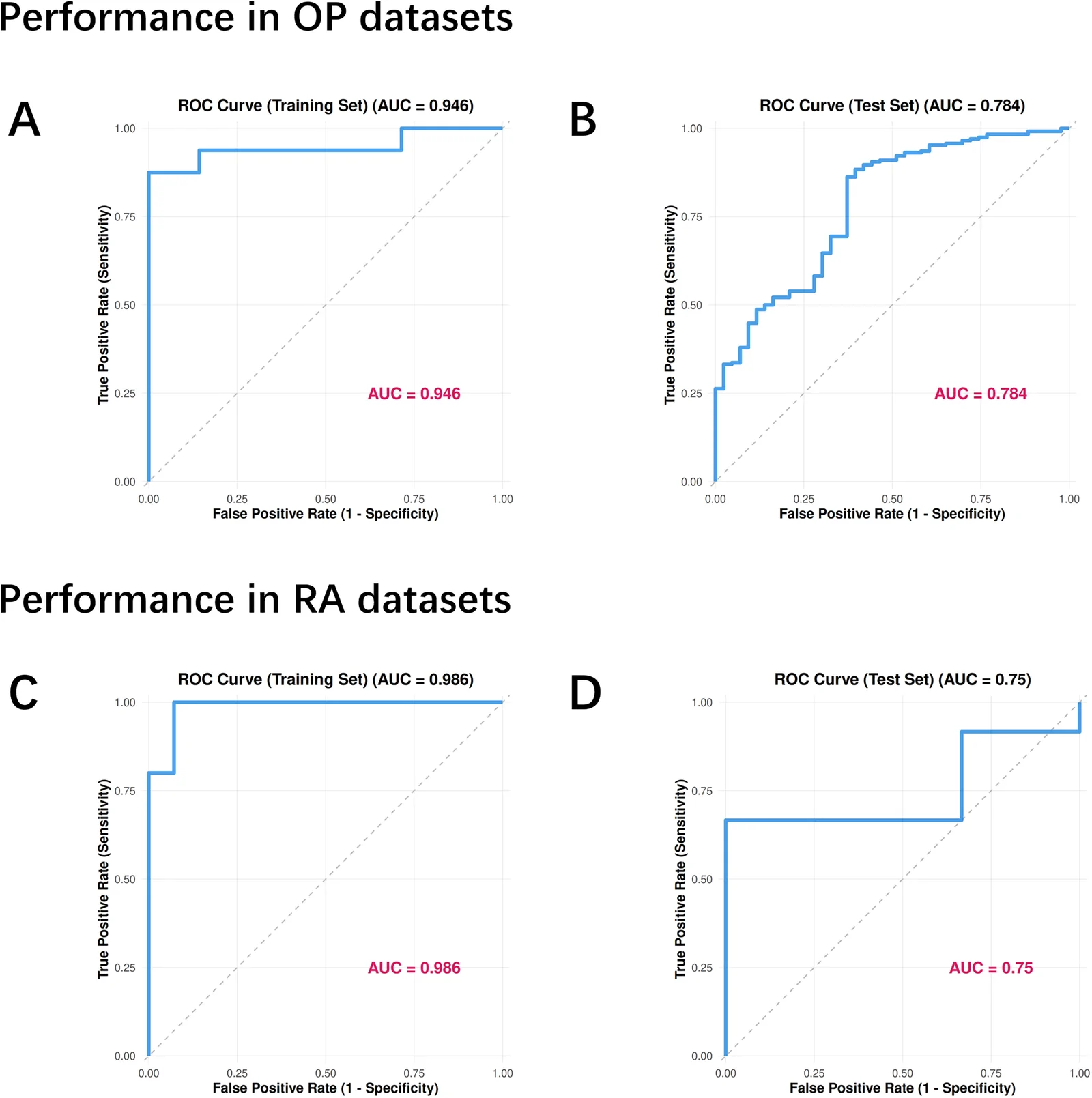
Construction and external validation of the multi-gene SVM diagnostic model for OP and RA. (A) Performance in the training set (GSE35959) using SVM. (B) Performance in the Test set (GSE230665) using SVM. (C) Performance in the training set (GSE77298) using SVM. (D) Performance in the Test set (GSE93272) using SVM.

To explore its relevance to RA, the same modeling pipeline was applied using GSE77298 for training and GSE93272 for external validation, with the original 3 hub genes retained as input features. The corresponding AUC were 0.986 and 0.75, respectively (Fig 4C, 4D), underscoring the model’s potential applicability across diseases with shared molecular features.

### Immune cell infiltration and correlation with shared hub genes

The enrichment analysis revealed that immunity plays a pivotal role in the development of both OP and RA. Therefore, we examined whether distinct immune infiltration patterns could be observed across 20 immune cell types using the CIBERSORT algorithm. First, we evaluated the immune cell composition in the bone tissue samples of OP dataset (GSE35959) and the synovial tissue samples of RA dataset (GSE77298) compared with their respective control groups. As shown in the violin plots, plasma cells showed a mild elevation, whereas the proportions of cytotoxic CD8 ⁺ T cells and activated NK cells were relatively unchanged in OP sample (Fig 5A). By contrast, the RA samples demonstrated an activated inflammatory immune signature (Fig 5B). B cells memory, macrophages M1 and plasma cells were markedly increased, while B cell naïve, dendritic cells resting, mast cells resting, NK cells resting and T cells CD4 memory resting were significantly reduced.

**Fig 5:**
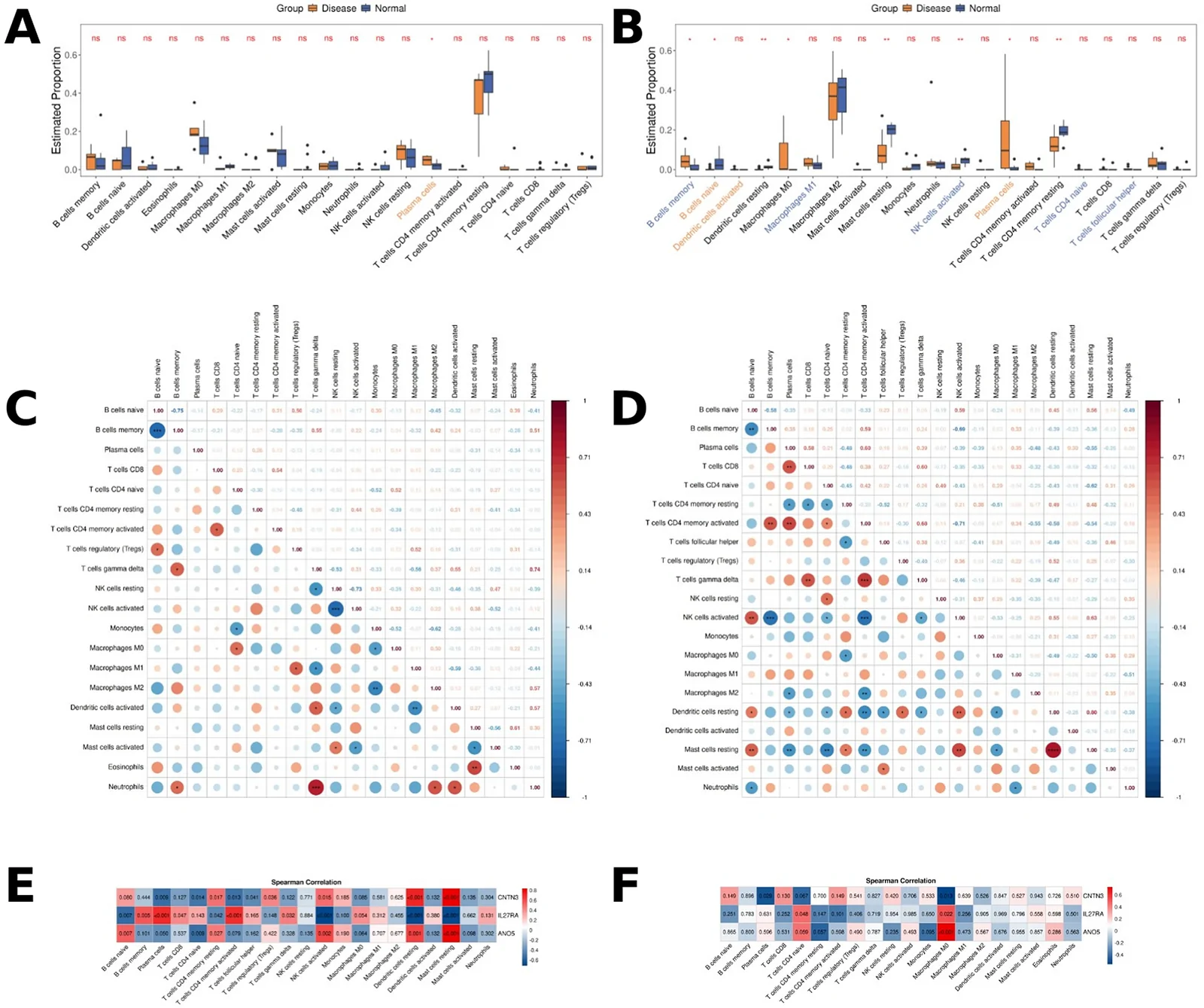
Immune cell infiltration landscapes and their correlation with shared hub genes in OP and RA. (A) Boxplots showing the relative proportions of 22 infiltrating immune cell types between disease and normal samples in OP, estimated using the CIBERSORT algorithm. (B) Boxplots showing the relative proportions of immune cell subsets in RA. Statistical significance was evaluated using the Wilcoxon rank-sum test, with P values annotated above each comparison (ns, not significant; P  <  0.05, *; P  <  0.01, **; P  <  0.001, ***). (C) Spearman correlation matrix depicting pairwise relationships among infiltrating immune cell types in OP. (D) Spearman correlation matrix of immune cell subsets in RA. Circle size and color intensity indicate the strength and direction of correlations. (E) Spearman correlation analysis between key feature genes and immune cell infiltration levels in OP. (F) Spearman correlation analysis between feature genes and immune cell subsets in RA.

The correlation analysis among infiltrating immune cells in OP samples (Fig 5C) revealed a network of associations. A positive correlation was observed between activated CD4 ⁺ memory T cells and CD8 ⁺ T cells. T cells regulatory (Tregs) showed a positive correlation with naive B cells. Gamma delta T cells exhibited a positive relationship with memory B cells but a negative correlation with resting NK cells. Regarding innate immune cells, M0 macrophages correlated positively with naïve B cells but negatively with monocytes. M1 macrophages showed a positive association with Tregs. Conversely, M2 macrophages were inversely correlated with total macrophage abundance. Neutrophils showed positive correlations with memory B cells, gamma delta T cells, M2 macrophages, and activated dendritic cells. The RA sample analysis (Fig 5D) showed a denser, more complex network of immune cell correlations. Strong positive associations were evident among activated CD4 ⁺ memory T cells, memory B cells, and plasma cells. CD8 ⁺ T cells were positively correlated with plasma cells and gamma delta T cells. Conversely, resting CD4 ⁺ memory T cells and naïve B cells displayed negative correlations with these activated cell populations. Activated NK cells exhibited opposite trends, showing a positive correlation with naïve B cells but a negative correlation with memory B cells. Macrophage subsets displayed distinct patterns: M0 and M2 macrophages were generally negatively correlated with activated T and B cells. Furthermore, dendritic cells and mast cells showed positive associations with several resting immune subsets but negative associations with activated lymphocytes.

Subsequently, we evaluated the correlation between the overall expression of CNTN3, IL27RA, and ANO5 and the abundance of various immune cell subsets in the OP and RA samples, using Spearman’s correlation analysis (Fig 5E and 5F). Only correlations with a P value < 0.05 are reported. In the OP dataset (Fig 5E), the expression of CNTN3 was positively correlated with T cells CD4 memory resting, and Tregs. NK cells activated and Dendritic cells resting, while negatively correlated with Plasma cells, T cells CD4 naive, T cells CD4 memory activated and T cells follicular helper. For IL27RA expression, positive correlations were observed with B cells memory, Plasma cells, T cells CD8, T cells CD4 memory activated and T cells gamma delta. Conversely, IL27RA displayed negative correlations with B cells naive, T cells CD4 memory resting, NK cells activated, Dendritic cells resting and Mast cells resting. Furthermore, ANO5 expression demonstrated positive correlations with B cells naive, T cells CD4 memory resting, NK cells activated, Dendritic cells resting and Mast cells resting. However, ANO5 was negatively correlated with Plasma cells and T cells CD4 naive. In the RA dataset (Fig 5F), the expression of CNTN3 was positively correlated with T cells CD8, T cells CD4 naive, T cells CD4 memory resting, Tregs, T cells follicular helper, Monocytes, Dendritic cells resting, and Dendritic cells activated. Conversely, CNTN3 was negatively correlated with Plasma cells, NK cells resting, Macrophages M0, Macrophages M1, and Macrophages M2. For IL27RA expression, a positive correlation was identified with Plasma cells, T cells CD8, T cells CD4 naïve, T cells CD4 memory resting, Tregs, T cells follicular/helper, Macrophages M0, Macrophages M1, Macrophages M2, and Dendritic cells resting. However, IL27RA showed negative correlations with NK cells activated, Monocytes, and Dendritic cells activated. Furthermore, ANO5 expression demonstrated positive correlations with T cells CD8, T cells CD4 naïve, Tregs, T cells follicular/helper, Macrophages M0, Macrophages M1, and Dendritic cells activated. In contrast, ANO5 was negatively correlated with Plasma cells, T cells CD4 memory resting, NK cells resting, NK cells activated, Monocytes, and Dendritic cells resting.

### Single-cell analysis for the location of hub genes

To comprehensively profile the cellular microenvironments associated with OP and RA, we performed scRNA-seq analysis on dataset derived from OP bone tissue (GSE147287) and RA synovial tissue (GSE200815). We used the SingleR package to annotate cell populations across two datasets, dividing the OP dataset into 13 cell clusters (Fig 6A). The OP bone tissue analysis revealed a high degree of cellular heterogeneity, including 277 Tissue stem cells, 1771 Osteoblasts, 1315 Neutrophils, 328 Monocytes, 879 Macrophages, 2299 various progenitors (HSC-G-CSF, GMP, CMP, BM & Prog.), 161 Fibroblasts, 411 T cells, and 290 B-cells. Results showed the complex interplay between immune, mesenchymal, and progenitor lineages in the bone microenvironment. We classified the RA dataset into 5 cell clusters (Fig 6B), including 258 Chondrocytes, 691 Endothelial cells, 1163 Fibroblasts, 807 Macrophages, and 428 T cells. Results showed Macrophages and Fibroblasts may play important roles of these cells as major mediators of synovial pathology.

**Fig 6:**
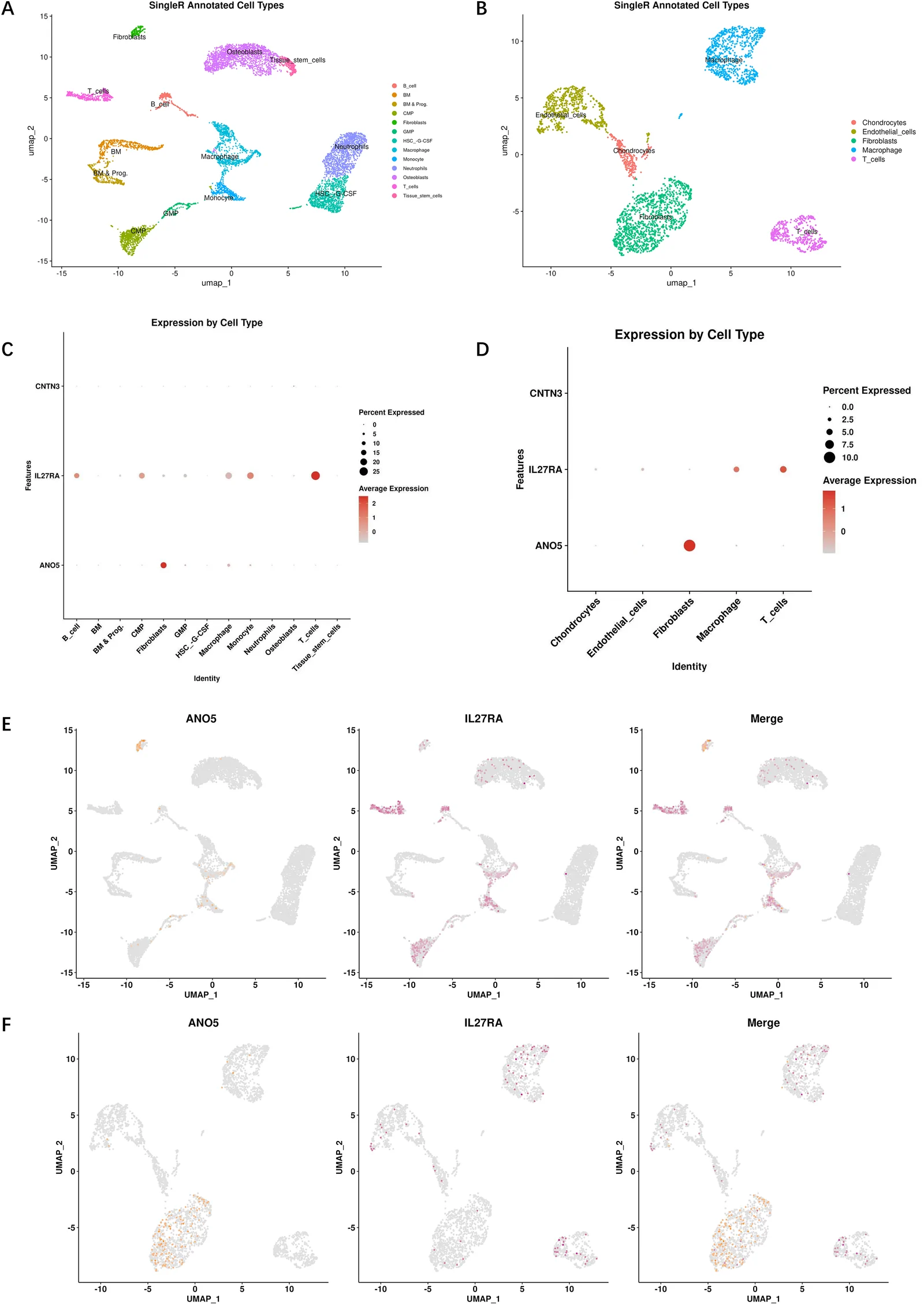
Single-cell transcriptomic profiling and cellular localization of hub genes in OP bone and RA synovium. (A) UMAP visualization of the 8219 cells in the OP single-cell RNA seq dataset GSE147287; Different colors indicate distinct clusters. (B) UMAP visualization of the 3347 cells in the RA single-cell RNA seq dataset GSE200815; (C) Dot plot illustrating the expression of ANO5 and IL27RA across annotated cell types in the OP dataset, with dot size representing the percentage of cells expressing the gene and color intensity indicating scaled average expression level. (D) Dot plot depicting ANO5 and IL27RA expression by cell type in the RA dataset, where dot size corresponds to percent expressed and color reflects scaled average expression, highlighting cell-type-specific patterns. (E) UMAP embeddings of OP cells colored by ANO5 and IL27RA expression levels, demonstrating the spatial distribution and intensity of expression within the cellular landscape. (F) UMAP embeddings of RA cells colored by ANO5 and IL27RA expression, illustrating regions of high and low expression across cell clusters.

We next investigated the cell type-specific expression of the highest diagnostic efficacy hub genes ANO5, IL27RA and CNTN3. In the OP bone tissue (Fig 6C), the Dot Plot indicated strong ANO5 expression primarily enriched in the Macrophage and Fibroblast populations, while IL27RA expression, quantified by the percentage of positive cells, was highest in T cells. In the RA synovial tissue (Fig 6D), Macrophages and Fibroblasts once again exhibited the highest mean expression level of ANO5. CNTN3 was expressed at negligible or undetectable levels across all cell types in both the OP and RA single-cell datasets. Given its minimal expression, CNTN3 was excluded from further cell-type-specific spatial analysis in the subsequent UMAP visualizations. Visualization of gene expression across the UMAP projections further confirmed the cellular specificities (Fig 6E, 6F). In the OP bone tissue (Fig 6E), ANO5 expression was distinctly localized to the clusters corresponding to the Macrophage/Fibroblast lineage, while IL27RA expression was primarily enriched in T cell, B cell, Monocyte, Macrophage and CMP. This precise enrichment pattern was replicated in the RA synovial tissue (Fig 6F), where ANO5 again marked the Macrophage/Fibroblast clusters and IL27RA was primarily enriched in T cell and Macrophage. T cell clusters. Collectively, these results establish macrophages and fibroblasts as the principal source of ANO5 expression, and T cells as the dominant cells expressing IL27RA, within the pathological microenvironments of both OP bone and RA synovium.

### Discussion

Immune and inflammatory disorders are commonly observed in individuals with OP [19–21] and growing evidence highlights a significant overlapping and synergistic interaction between OP and RA [22–24]. RA, a chronic autoimmune disorder, has been always considered to induce bone loss, thereby increasing susceptibility to OP and the incidence of fractures and skeletal complications. Both conditions share systemic inflammation as a key pathogenic hallmark, with RA potentially exacerbating bone resorption and accelerating the progression of OP [25]. Contributing factors such as chronic glucocorticoid therapy, advanced age, female sex, sedentary lifestyle, and elevated inflammatory biomarkers have been identified as risk factors for OP in patients with RA [26]. However, most of these associations have been established through clinical and serological observations, while the molecular mechanism remains to be further research. Conventional treatment modalities, such as Bisphosphonates, are effective in increasing bone mineral density but insufficient in suppressing immune-mediated inflammatory osteoclastic activity [27]. Conversely, disease-modifying antirheumatic drugs (DMARDs) can alleviate joint inflammation; however, their efficacy in preserving bone mass remains inconsistent [28]. They have shown limited effectiveness in addressing the dual pathology, underscoring the need for innovative therapeutic approaches that target specific molecular and genetic pathways. Furthermore, the involvement of multiple organ systems and heterogeneous cellular populations in the pathophysiology of both RA and OP presents substantial challenges to the development of targeted therapeutic strategies.

Recent multi‑omics studies have increasingly highlighted the value of integrating transcriptomic, epigenomic, and proteomic data to dissect the complex molecular networks underlying musculoskeletal diseases [29]. These approaches have proven particularly effective in identifying robust immune‑related gene signatures that correlate with disease activity and treatment response. In parallel, the characterization of immune cell infiltration patterns using deconvolution algorithms has emerged as a powerful strategy to link peripheral immune dysregulation with local tissue pathology [30]. Our CIBERSORT‑based findings, which revealed coordinated yet distinct immune landscapes in OP bone and RA synovium, are consistent with these emerging paradigms. Furthermore, the concept of osteoimmunology—the bidirectional crosstalk between the immune and skeletal systems—has gained substantial traction, with growing evidence that shared inflammatory pathways, including cytokine signaling and RANKL‑mediated osteoclastogenesis, underpin the comorbidity of inflammatory and metabolic bone diseases [31]. Our identification of ANO5 and IL27RA as shared candidates linking immune cell populations (macrophages, T cells) to bone‑related processes aligns with this osteoimmune framework and provides specific molecular candidates for future mechanistic investigation.

In this study, we aimed to determine whether OP and RA share common molecular and genetic mechanisms by conducting integrative bioinformatics analyses combined with machine learning algorithms. Initially, we identified coexpression modules shared between OP and RA. To improve the accuracy of gene selection, we utilized WGCNA-derived module genes, resulting in 329 shared candidate genes. Notably, the relatively low overlap (10%) at the individual gene level underscores that the shared molecular architecture between OP and RA is primarily manifested at the pathway and network levels—a pattern commonly observed in complex polygenic diseases—rather than at the level of individual diagnostic markers. Enrichment analysis showed that these genes were strongly related to muscle development, cytokine signaling, and inflammatory pathways. These results support the view that musculoskeletal dysfunction and chronic inflammation act as shared pathogenic axes contributing to bone loss in both diseases. Subsequently, machine learning approaches were employed to identify robust hub genes. The Random Forest revealed three shared hub genes—ANO5 and IL27RA, along with CNTN3 as a computationally nominated candidate—which exhibited strong diagnostic potential with high AUC values. While each of these genes holds value as a potential biomarker, relying on individual genes for diagnosis may not fully capture the complexity of RA and OP comorbidity. Therefore, we constructed a diagnostic model using the SVM algorithm to improve diagnostic accuracy. The model showed strong predictive performance in both training and validation cohorts, supporting the utility of these genes in forecasting RA and OP comorbidity.

To further explore the biological relevance of these hub genes, we performed CIBERSORT-based immune cell infiltration analysis. We found evidence of coordinated immune regulation in both conditions. Activated T cells, B cells, and plasma cells showed strong positive associations in RA, consistent with synovial hyperactivity. Macrophage subsets were inversely correlated with activated lymphocytes, suggesting dynamic roles in inflammation and tissue repair. These correlation patterns suggest potential cellular interactions that may contribute to local bone remodeling and joint pathology. Furthermore, the hub genes ANO5 and IL27RA were significantly associated with immune cell infiltration. IL27RA correlated with activated T cells and macrophages, suggesting a role in regulating inflammatory cascades and T cell differentiation. ANO5 showed consistent associations with macrophage populations and was primarily expressed in macrophages and fibroblasts, indicating a potential role in membrane repair or matrix interactions. ANO5 was highly expressed in macrophages and fibroblasts, both central regulators of tissue remodeling. IL27RA was enriched in T cells, aligning with its known immunomodulatory function. By contrast, CNTN3 showed minimal enrichment at the single-cell level, suggesting a limited role in dominant inflammatory cell populations, while indirect involvement through signaling or structural interactions remains possible. These cell-type specific patterns provide biological support for their contribution to OP and RA.

IL-27RA partners with glycoprotein 130 to form a heterodimeric signaling complex. This complex is responsible for transducing signals initiated by its ligand, the heterodimeric cytokine IL-27 (composed of p28 and EBI3) [32]. IL27RA encodes the receptor subunit that, together with gp130, forms the IL‑27 receptor complex. While the ligand IL‑27 has been demonstrated to play a critical role in bone homeostasis——promoting osteoblast differentiation and suppressing osteoclastogenesis——our data only establish IL27RA transcript expression in specific cell types (T cells and macrophages). Whether IL27RA expression correlates with functional IL‑27 signaling activity in OP or RA tissues cannot be determined from the present dataset. Nevertheless, the cell‑type‑specific expression of IL27RA in T cells and macrophages aligns with its known immunomodulatory functions and supports its potential relevance to the immuno‑skeletal crosstalk underlying both diseases [33]. Additionally, it has been demonstrated that IL‑27 upregulates key genes, including STAT1, STAT4, and CXCL11, thereby activating anti‑tumor and immunomodulatory pathways and enhancing the immunogenicity of the tumor microenvironment. In parallel, by enriching signaling pathways associated with osteoclast differentiation, immune response, and inflammatory regulation, IL‑27 synergizes with the pro‑osteogenic properties of cabo [34]. In human osteoclastogenesis, IL‑27 suppresses RANKL‑induced osteoclastogenesis by downregulating the expression of NFATc1, RANK, and TREM‑2 and inhibiting the RANK signaling pathway, thereby reducing osteoclast formation and activity [35]. IL27RA is also critically involved in the development of RA. Research indicates that IL27RA is primarily expressed in T‑lymphocytes of RA patients, and its expression shows a positive correlation with plasma cytokines, tissue TNF, and synovial markers of B‑cell‑driven inflammation (such as IL17A, CD21L), implying that the IL‑27/IL27RA pathway may contribute to the regulation of B‑cell‑dominant synovial inflammation [36]. A previous study has demonstrated that in RA, IL-27 directly activates patient-derived fibroblast-like synoviocytes, inducing STAT1-dependent upregulation of adhesion molecules (ICAM-1, VCAM-1) and pro-inflammatory mediators (IL-6, CXCL10). Additionally, IL-27 synergizes with TNF-α to amplify synovial inflammation and joint damage [37].

ANO5 (anoctamin 5) is a calcium-activated chloride channel that plays a crucial role in maintaining intracellular calcium homeostasis, thereby regulating bone cell survival, differentiation, and function. Emerging evidence indicates that ANO5 is particularly critical for osteoclast biology. Loss-of-function mutations in ANO5 disrupt calcium ion homeostasis, leading to excessive endoplasmic reticulum stress (ERS) and activation of the CHOP signaling pathway, which markedly increases osteoclast apoptosis and impairs their survival [38]. Consistently, ANO5 deficiency or mutation significantly inhibits osteoclast differentiation and function by suppressing RANKL-induced early signaling events and downregulating key osteoclastogenic transcription factors, including NFATc1 [39]. In contrast to its relatively well-defined role in osteoclasts, the function of ANO5 in osteoblasts remains context-dependent and somewhat controversial. Several studies suggest that ANO5 may negatively regulate osteoblast activity. For example, Ano5 knockout enhances the osteogenic potential of mouse calvarial osteoblasts through activation of ATG9A-dependent autophagy pathways, and aberrant activation of the miR-34c-5p/KLF4/β-catenin axis has been shown to promote osteoblast function in Ano5-deficient mouse models [39, 40]. Conversely, other studies support a pro-osteogenic role for ANO5, demonstrating that ANO5 maintains osteoblast function by regulating intracellular calcium signaling. ANO5 deficiency leads to attenuated calcium transients, reduced Wnt/β-catenin signaling, impaired alkaline phosphatase activity, and diminished matrix mineralization capacity, partly through disruption of Akt signaling pathways, as observed in both knockout and mutant (ANO5^Cys360Tyr^) mouse models [41, 42]. To our knowledge, ANO5 has not been directly investigated in the context of RA synovitis or bone erosion. Our computational findings suggest a potential link that warrants future functional exploration, but no causal or mechanistic role can be inferred from the present data.

CNTN3 (Contactin 3), a neural adhesion molecule primarily expressed in the central nervous system, was identified as a computationally nominated candidate by our machine learning pipeline. However, comprehensive literature searches reveal no direct evidence linking CNTN3 to bone homeostasis, osteoclastogenesis, osteoblast function, or inflammatory processes in OP or RA. Moreover, our single-cell data showed negligible expression of CNTN3 across major cell types in OP bone and RA synovial tissues, and its correlations with immune subsets were limited. While we cannot entirely exclude an indirect role—possibly through unexplored neuro-immune or neuro-musculoskeletal crosstalk—the current evidence does not support CNTN3 as a primary mediator in OP-RA shared pathology. Functional investigations are warranted to clarify whether CNTN3 has any mechanistic relevance to these diseases.

Despite the strengths of this integrative analytical framework, several limitations should be acknowledged. First, the present study was based on publicly available transcriptomic datasets, and the absence of datasets specifically representing RA–OP comorbidity limited causal inference between the two diseases. Second, although machine learning and single-cell analyses strengthened the robustness of our findings, experimental validation in vitro and in vivo is still required to confirm the functional roles of the identified hub genes. Third, tissue heterogeneity and differences between peripheral blood, bone, and synovial microenvironments may influence gene expression patterns. Fourth, the sample sizes of both the training and validation cohorts for OP and RA are relatively modest, and the heterogeneity inherent in datasets derived from different platforms may compromise the generalizability of our findings. Consequently, the conclusions of the present study warrant further confirmation in larger, more homogeneous prospective cohorts. Fifth, the CIBERSORT algorithm was originally developed for blood-based transcriptomic deconvolution, and its application to bone and synovial tissues—which have distinct cellular compositions and matrix environments—may introduce biases. Therefore, our immune infiltration results should be interpreted as correlative rather than definitive, and warrant validation using targeted approaches such as flow cytometry or multiplex immunohistochemistry in future studies. Sixth, the sample sizes of the training cohorts, particularly the OP training set (GSE35959, n = 5 OP cases), are relatively small, which increases the risk of overfitting and limits statistical power. Although we sought to mitigate this limitation through external validation in independent cohorts and cross validation within the training set, the present findings should be interpreted as exploratory and hypothesis generating in nature. Larger, well powered prospective cohorts are warranted to confirm the diagnostic utility of the proposed gene signature. Moreover, the training and validation datasets were derived from distinct platforms; while we deliberately refrained from cross dataset normalization to avoid information leakage, inherent technical heterogeneity between cohorts may have influenced the observed effect sizes. Future studies incorporating larger cohorts, comorbidity-specific samples, and functional experiments will be essential to further elucidate the immuno-skeletal mechanisms linking RA and osteoporosis and to translate these findings into clinical applications.

## Conclusion

In conclusion, this study systematically identified shared molecular signatures underlying osteoporosis and rheumatoid arthritis through integrative bioinformatics and machine learning approaches. ANO5 and IL27RA emerged as the most robust candidate hub genes associated with immune regulation and bone remodeling, while CNTN3—although identified by machine learning—should be interpreted with caution due to limited supporting evidence. Collectively, these findings provide novel insights into the immuno-skeletal crosstalk between RA and OP and may facilitate the development of improved diagnostic markers and targeted therapeutic strategies for their comorbidity.

## Supporting information

S1 Fig The resulting network comprised 233 nodes and 375 edges. (TIF)

S2 Fig The top5 significant genes with the highest ranking. (TIF)

S1 Table The demographic data of samples in related data sets. (DOCX)
