# Supplementary material for: Integrative analysis reveals shared biomarkers and pathways in osteoporosis and rheumatoid arthritis using machine learning and single-cell sequencing
Source: PLoS One. 2026 Nov 4;21(11):e0362219. doi: 10.1371/journal.pone.0362219 (PMC13734575; doi:10.1371/journal.pone.0362219)
Supplement: Supplementary file 1 [file pone.0362219.s003.docx]

Supplementary Table S1. Characteristics of datasets for rheumatoid arthritis (RA) and osteoporosis (OP) co-morbidity analysis

| **GEO datasets** | **Platform** | **Sample size** | |  |
| --- | --- | --- | --- | --- |
|  |  | **Patients** | **Controls** | |
| GSE35959(OP) | GPL570 | 5 | 14 | |
| GSE230665(OP) | GPL570 | 12 | 3 | |
| GSE147287(OP) | GPL24676 | 1 | 0 | |
| GSE77298(RA) | GPL570 | 16 | 7 | |
| GSE93272(RA) | GPL570 | 229 | 46 | |
| GSE200815(RA) | GPL24676 | 4 | 0 | |
